# Annotated image dataset of fire blight symptoms for object detection in orchards

**DOI:** 10.1016/j.dib.2024.110826

**Published:** 2024-08-10

**Authors:** Virginia Maß, Pendar Alirezazadeh, Johannes Seidl-Schulz, Matthias Leipnitz, Eric Fritzsche, Rasheed Ali Adam Ibraheem, Martin Geyer, Michael Pflanz, Stefanie Reim

**Affiliations:** a Leibniz Institute for Agricultural Engineering and Bioeconomy^1^, Department Agromechatronics, Potsdam, Germany; b geo-konzept^1^, Gesellschaft für Umweltplanungssyteme mbH, Adelschlag, Germany; c Julius Kühn-Institute^1^, Federal Research Centre for Cultivated Plants, Institute for Breeding Research on Fruit Crops, Dresden, Germany

**Keywords:** YOLO, *Erwinia amylovora*, Disease monitoring, Phenotyping, Machine learning

## Abstract

The monitoring of plant diseases in nurseries, breeding farms and orchards is essential for maintaining plant health. Fire blight (*Erwinia amylovora*) is still one of the most dangerous diseases in fruit production, as it can spread epidemically and cause enormous economic damage. All measures are therefore aimed at preventing the spread of the pathogen in the orchard and containing an infection at an early stage [1–6]. Efficiency in plant disease control benefits from the development of a digital monitoring system if the spatial and temporal resolution of disease monitoring in orchards can be increased [7]. In this context, a digital disease monitoring system for fire blight based on RGB images was developed for orchards.

Between 2021 and 2024, data was collected on nine dates under different weather conditions and with different cameras. The data source locations in Germany were the experimental orchard of the Julius Kühn Institute (JKI), Institute of Plant Protection in Fruit Crops and Viticulture in Dossenheim, the experimental greenhouse of the Julius Kühn Institute for Resistance Research and Stress Tolerance in Quedlinburg and the experimental orchard of the JKI for Breeding Research on Fruit Crops located in Dresden–Pillnitz. The RGB images were taken on different apple genotypes after artificial inoculation with *Erwinia amylovora*, including cultivars, wild species and progeny from breeding.

The presented ERWIAM dataset contains manually labelled RGB images with a size of 1280  × 1280 pixels of fire blight infected shoots, flowers and leaves in different stages of development as well as background images without symptoms. In addition, symptoms of other plant diseases were acquired and integrated into the ERWIAM dataset as a separate class. Each fire blight symptom was annotated with the Computer Vision Annotation Tool (CVAT [8]) using 2-point annotations (bounding boxes) and presented in YOLO 1.1 format (.txt files). The dataset contains a total of 1611 annotated images and 87 background images. This dataset can be used as a resource for researchers and developers working on digital systems for plant disease monitoring.

Specifications Table

**Table:** 

Subject	Computer Science; Computer Vision and Pattern Recognition
Specific subject area	The dataset contains resized and annotated RGB image files of fire blight infected leaves, shoots and flowers, as well as symptoms of diseases similar in appearance with symptoms of fire blight, from the experimental orchard and greenhouse for computer vision and pattern recognition applications.
Type of data	Analysed, filtered and processed digital 2D RGB image files (.JPG). Size of the RGB images: 3 × 1280 × 1280 pixels. YOLO 1.1 annotation files [8] (.txt) Table of the image metadata [9] (.xlsx) Table of the annotation metadata (.xlsx)
Data collection	The raw RGB images were collected using the cameras: Smartphone Samsung [10,11], Tablet Samsung [12], Canon EOS 70D [13] and Canon EOS 90D [14]. The annotation tool CVAT [8] was used for the 2-point bounding box annotation. The images were resized to 1280 × 1280 pixels with the help of the Python library Pillow using the Lanczos filter [15].
Data source location	The data source location was the experimental orchard of the Julius Kühn-Institute (JKI - Federal Research Centre for Cultivated Plants) at the Institute for Plant Protection in Fruit Crops and Viticulture in Dossenheim (Germany) located in Kirschgartshausen [49°35ʹ04″N 8°26ʹ46″E], the experimental greenhouse of the JKI for Resistance Research and Stress Tolerance located in Quedlinburg (Germany) [51°46ʹ22″N 11°08ʹ41″E] and at the experimental orchard of the JKI for Breeding Research on Fruit Crops located in Dresden-Pillnitz (Germany) [51°00ʹ01″N 13°53ʹ12"E].
Data accessibility	Repository name: Mendeley Data Data identification number: 10.17632/fpmnncmg84.1 Direct URL to data: https://data.mendeley.com/datasets/fpmnncmg84/1

## 1 Value of the Data

• In the experimental greenhouse of the JKI-Quedlinburg Institute, around 2000 different genotypes of apple breeding material were artificially inoculated with *Erwinia amylovora* in 2021 and 2022, which could be used to record fire blight symptoms. The JKI-Dossenheim Institute has a heterogeneous apple orchard consisting of 600 to 700 trees mainly ‘Gala’ but also single trees of other apple varieties and apple wild species. Every year, one third of the orchard with the oldest trees was used for fire blight inoculation. Artificial inoculation took place four to six weeks before the recording period. The high genetic diversity captured in the RGB images of the ERWIAM dataset can help to utilise the responses of different genotypes to the fire blight disease for the application of deep learning methods and is therefore important for the scientific community.
• Plant breeders and scientists can use the ERWIAM dataset to test and refine object detection using the model pathogen *Erwinia amylovora* in precision agriculture and plant disease monitoring.
• Digital monitoring should contribute to the early identification of the phenotypic characteristics of pathogens and facilitate the targeted use of plant protection products.

## 2 Background

Global trade and changing climatic conditions mean that immigrating pests and diseases are increasingly able to establish themselves in Europe. *Erwinia amylovora* was introduced to Europe in the 1950s and has spread throughout most of Europe since around 2006 [3,16]. If it spreads uncontrollably, fire blight can cause considerable economic damage to orchards. Although an infestation can be controlled by the use of antibiotics such as streptomycin, its use is banned or strictly regulated in most European countries [2,5,6]. Early detection of the first symptoms of fire blight and localisation of the diseased trees in the orchard are important in order to be able to apply the containment methods in a controlled and timely manner. The simple handling, the cost-effective availability and the system-integration of high-resolution RGB cameras are advantages compared to cameras with other sensors [7]. Since no comparable dataset of RGB images with the size of 1280 × 1280 pixels and annotations was available open-source, which included shoot, flower and leaf symptoms as well as symptoms similar to fire blight, the ERWIAM dataset was created. The development of a digital method for detecting fire blight symptoms in orchards can be an effective method for monitoring this pathogen. In order to utilise the possibilities of monitoring and mapping with RGB images, it is essential to create a dataset with the specific symptoms of the model pathogen *Erwinia amylovora*.

## 3 Data Description

The aim was to visualise the diversity of fire blight symptoms in apple genotypes that occurred in greenhouse and the field. The experimental orchard of the Julius Kühn-Institute (JKI - Federal Research Centre for Cultivated Plants) at the Institute for Plant Protection in Fruit Crops and Viticulture in Dossenheim (Germany) located in Kirschgartshausen, the experimental greenhouse of the JKI for Resistance Research and Stress Tolerance located in Quedlinburg (Germany) and at the experimental orchard of the JKI for Breeding Research on Fruit Crops located in Dresden–Pillnitz (Germany) served as the data source. 1698 RGB images with different resolutions (Table 1) were used to create the ERWIAM dataset.

**Table 1:** Camera models used with the different resolutions, image sizes, focal lengths and the range of light values of the collected RGB images.

Camera model	Sensor type	Focal length [mm]	Focal length 35 mm format [mm]	Image width [px]	Image height [px]	Image resolution [MPixel]
Canon EOS 90D [14]	CMOS	18.0 to 55.0	29.1 to 88.9	6960	4640	32.29
Canon EOS 70D [13]	CMOS	18.0 to 55.0	50.4 to 86.7	5472	3648	19.96
Samsung tablet [12]	CMOS	2.9	28	3264	2448	7.99
Samsung smartphone A [11]	CMOS	5.4 to 7.1	26 to 76	4032	3024	12.19
		1.7	13	4000	3000	12.00
Samsung smartphone B [10]	ISOCELL	4.3	26	4032	3024	12.19

**Fig. 1:**
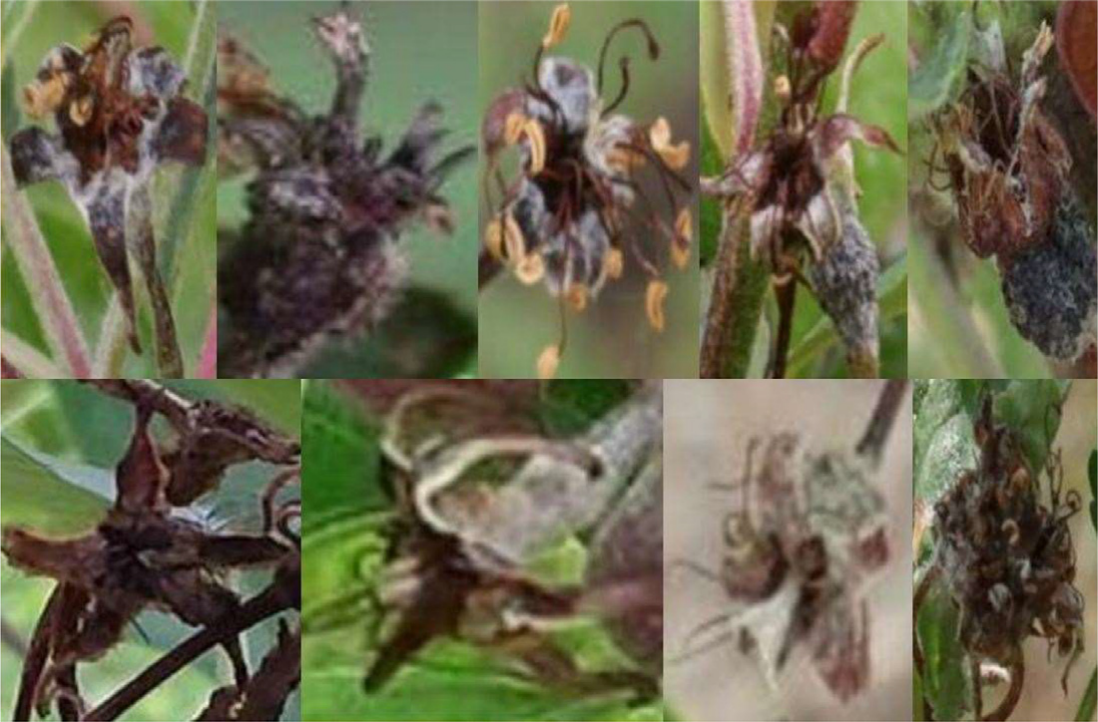
Example of images with fire blight infected flowers used for the annotation of the class “FLOWER” in the ERWIAM dataset. Images were taken in the experimental orchard in Kirschgartshausen.

The resulting 1611 original images were carefully checked for quality and the presence of disease symptoms in order to label them using 2-point annotation with the annotation tool CVAT [8]. A further 87 RGB images were added to the data set as background images that showed no fire blight symptoms. Rectangular bounding boxes were drawn around the symptomatic area. The flowers infected with *Erwinia amylovora* were labelled as the class ``FLOWER'' (Fig. 1).

**Fig. 2:**
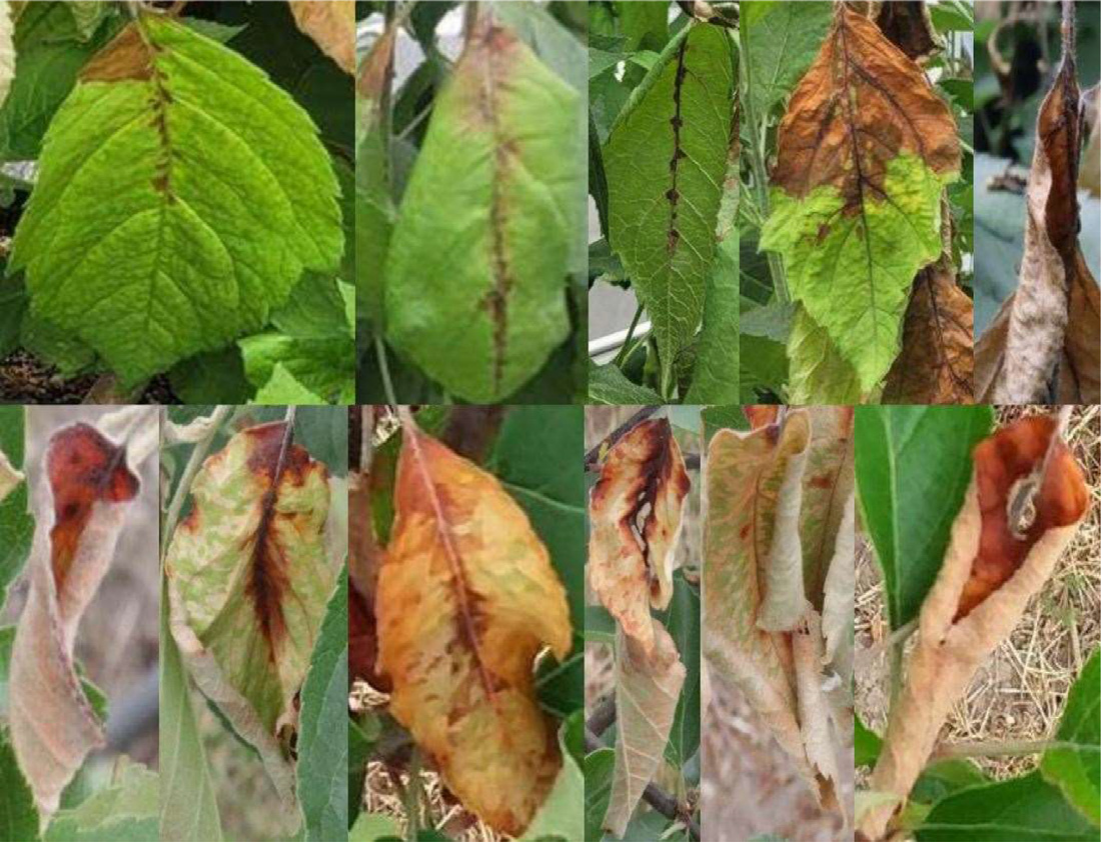
Example of images with fire blight infected leaves used for the annotation of the class “LEAF__” in the ERWIAM dataset. Images were taken in the experimental greenhouse in Quedlinburg (samples in the top row) and experimental orchard in Kirschgartshausen (samples in bottom row).

**Fig. 3:**
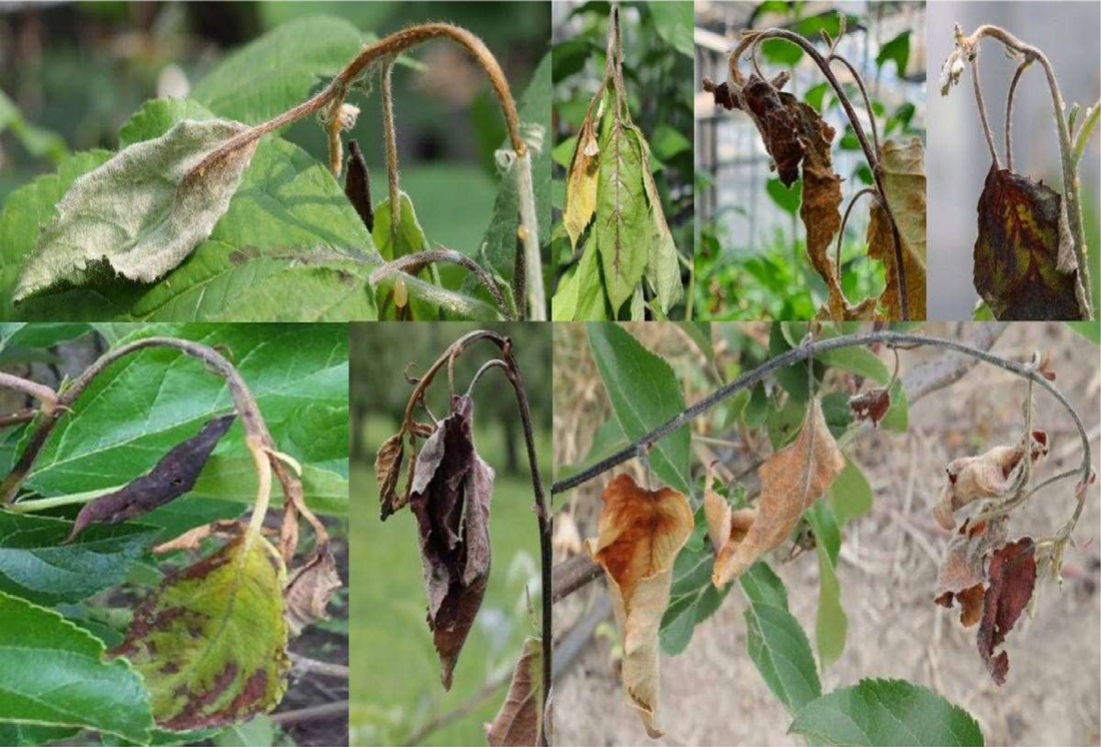
Example of images with fire blight infected shoots used for the annotation of the class “SHOOT_” in the ERWIAM dataset. Images were taken in the experimental greenhouse in Quedlinburg (samples in the top row) and experimental orchard in Kirschgartshausen (samples in bottom row).

Leaves with fire blight symptoms included several disease stages (Fig. 2) and were labelled as class ``LEAF__''. Fire blight symptoms on the shoot were included in the dataset as the class ``SHOOT_'' (Fig. 3).

**Fig. 4:**
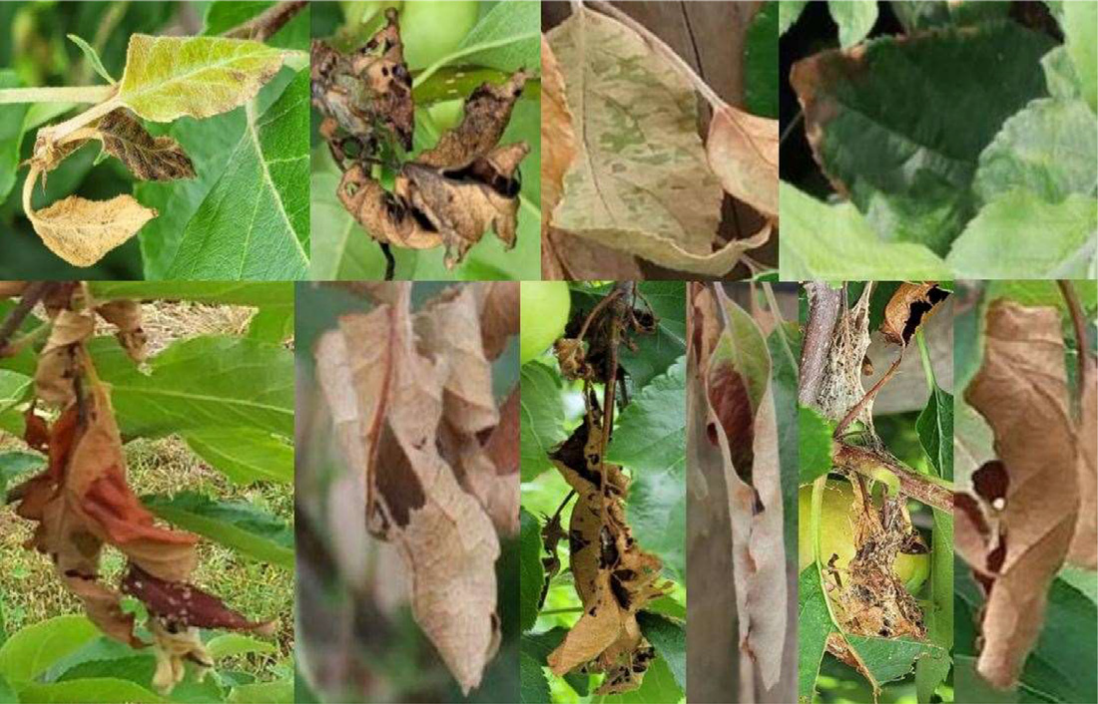
Example of images with symptoms of other plant diseases that look like fire blight symptoms used for the annotation of the class ``MAYBE_'' in the ERWIAM dataset. Images were taken in the experimental orchard in Kirschgartshausen.

In addition, symptoms of other plant conditions, as well as symptoms, that may be attributable to fire blight, but could not be clearly assigned based on RGB images, were also recorded and integrated into the ERWIAM dataset as a separate class ``MAYBE_'' (Fig. 4).

After the annotation work was completed, a labelling file (.txt) in YOLO 1.1 format was created for each image and used for model training. 15,761 annotations belonging to all classes were created manually on 1611 images (Table 2).

**Table 2:** Overview of all annotations in the ERWIAM dataset by date of recording, location and class.

Date	Location	Classes				Sum
		MAYBE_	FLOWER	LEAF__	SHOOT_	
26/05/2021	KGH^a^	0	238	1	0	239
27/05/2021	KGH^a^	0	19	0	0	19
09/06/2021	QLB^b^	441	0	555	501	1497
01/07/2021	KGH^a^	315	245	134	30	724
04/05/2022	QLB^b^	250	0	352	242	844
22/06/2022	KGH^a^	4754	4787	2450	447	12,438
Sum		5760	5289	3492	1220	**15,761**

a KGH: experimental orchard in Kirschgartshausen, JKI-Institute for Plant Protection in Fruit Crops and Viticulture in Dossenheim (Germany).

b QLB: experimental greenhouse in Quedlinburg, JKI- Institute for Resistance Research and Stress Tolerance in Quedlinburg (Germany).

**Fig. 5:**
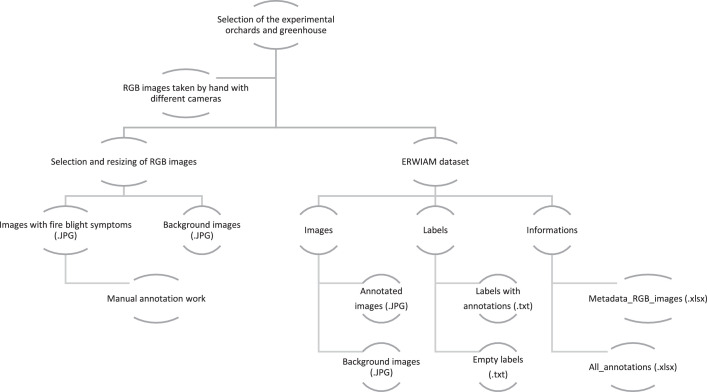
Data collection and folder structure of the ERWIAM dataset.

The following information can be found in the text files of the annotated images in the following order: class_ID, x_centre, y_centre, width, height. Each image file in the ERWIAM dataset has a corresponding text file in YOLO 1.1 format. The corresponding .txt files for each image are located in the labels folder. In addition to the images and the label folder, there is also an information folder for the ERWIAM dataset (Fig. 5). The information folder contains an All_annotations.xlsx file in TensorFlow Object Detection format, which lists all annotations with the values class, height, width, xmin, ymin, xmax, ymax for each file name. The metadata [9] of the images are stored as a Metadata_RGB_image.xlsx file in the information folder so that users can select or re-sort the dataset by camera models (Table 1), frequency of annotations (Table 2), locations and date (Table 3), camera settings (Table 4) or other parameters.

**Table 3:** Number of RGB images with a size of 1280 × 1280 pixels from the experimental orchards and the experimental greenhouse that were used for annotations and the use of background images.

Date	Location	Label	Number of images			Sum
			Canon EOS 70D and Canon EOS 90D	Samsung Tablet	Smartphone A and Smartphone B	
26/05/2021	KGH^a^	Annotated	50	61	0	111
27/05/2021	KGH^a^	Annotated	0	13	0	13
09/06/2021	QLB^b^	Annotated	8	11	32	51
01/07/2021	KGH^a^	Annotated	52	93	26	171
04/05/2022	QLB^b^	Annotated	46	0	0	46
22/06/2022	KGH^a^	Annotated	1049	170	0	1219
12/09/2022	DD^c^	Background	15	0	0	15
08/04/2023	DD^c^	Background	46	0	0	45
28/04/2023	DD^c^	Background	26	0	0	27
Sum			1292	348	58	**1698**

a KGH: experimental orchard in Kirschgartshausen, JKI-Institute for Plant Protection in Fruit Crops and Viticulture in Dossenheim (Germany).

b QLB: experimental greenhouse in Quedlinburg, JKI- Institute for Resistance Research and Stress Tolerance in Quedlinburg (Germany).

c DD: experimental orchard in Dresden-Pillnitz, JKI-Institute for Breeding Research on Fruit Crops in Dresden-Pillnitz (Germany).

**Table 4:** ERWIAM dataset overview with the RGB image and sensor metadata [9] from 2021 up to and including 2023 in the experimental orchards and in the experimental greenhouse.

Date	Location	Camera model	Light value [LV]	ISO	*F*-number	Shutter speed
26/05/2021	KGH^a^	Canon EOS 70D	+9.5 to +11.9	250 to 6400	10.0–14.0	1/49 to 1/256
		Samsung tablet	+9.1 to +12.4	40 to 64	1.9	1/100 to 1/606
27/05/2021	KGH^a^	Samsung tablet	+9.1 to +11.1	40 to 64	1.9	1/100 to 1/234
09/06/2021	QLB^b^	Canon EOS 70D	+6.8 to +9.0	320 to 6400	3.5 to 9.0	1/32 to 1/395
		Samsung tablet	+8.1 to +8.8	40 to 80	1.9	1/50 to 1/100
		Samsung smartphone A	+9.8 to +11.8	40 to 50	1.8 to 2.4	1/100 to 1/439
		Samsung smartphone B	+8.8 to +11.1	50 to 125	2.4	1/100 to 1/191
01/07/2021	KGH^a^	Canon EOS 70D	+9.9 to +10.6	400	5.6	1/128 to 1/197
		Samsung tablet	+7.9 to +13.7	40 to 80	1.9	1/33 to 1/838
		Samsung smartphone A	+9.0 to +12.8	25 to 64	1.8 and 2.4	1/100 to 1/632
04/05/2022	QLB^b^	Canon EOS 90D	+9.0 to +12.6	1600	10	1/80 to 1/1000
22/06/2022	KGH^a^	Canon EOS 90D	+11.9 to +13.6	400 to 800	5.6–7.1	1/1000
		Samsung tablet	+10.1 to +13.4	40	1.9	1/122 to 1/1203
12/09/2022	DD^c^	Canon EOS 90D	+12.7 to +14.7	100	9.0 to 13.0	1/320 to 1/640
08/04/2023	DD^c^	Canon EOS 90D	+12.6 to +14.0	100	5.6 to 7.1	1/200 to 1/320
28/04/2023	DD^c^	Canon EOS 90D	+12.0 to +13.3	100	5.0 to 6.3	1/160 to 1/250

a KGH: experimental orchard in Kirschgartshausen, JKI-Institute for Plant Protection in Fruit Crops and Viticulture in Dossenheim (Germany).

b QLB: experimental greenhouse in Quedlinburg, JKI- Institute for Resistance Research and Stress Tolerance in Quedlinburg (Germany).

c DD: experimental orchard in Dresden-Pillnitz, JKI-Institute for Breeding Research on Fruit Crops in Dresden-Pillnitz (Germany).

The annotated images were resized to 1280 × 1280 pixels in pre-processing by using the Python library Pillow [15] and saved in JPG format. In addition, 87 background images (1280 × 1280 pixels) without disease symptoms were added in JPG format (Table 3).

There is an empty .txt file in the label folder for each background image in the image folder that has no fire blight symptoms and therefore no annotations (Fig. 5).

**Fig. 6:**
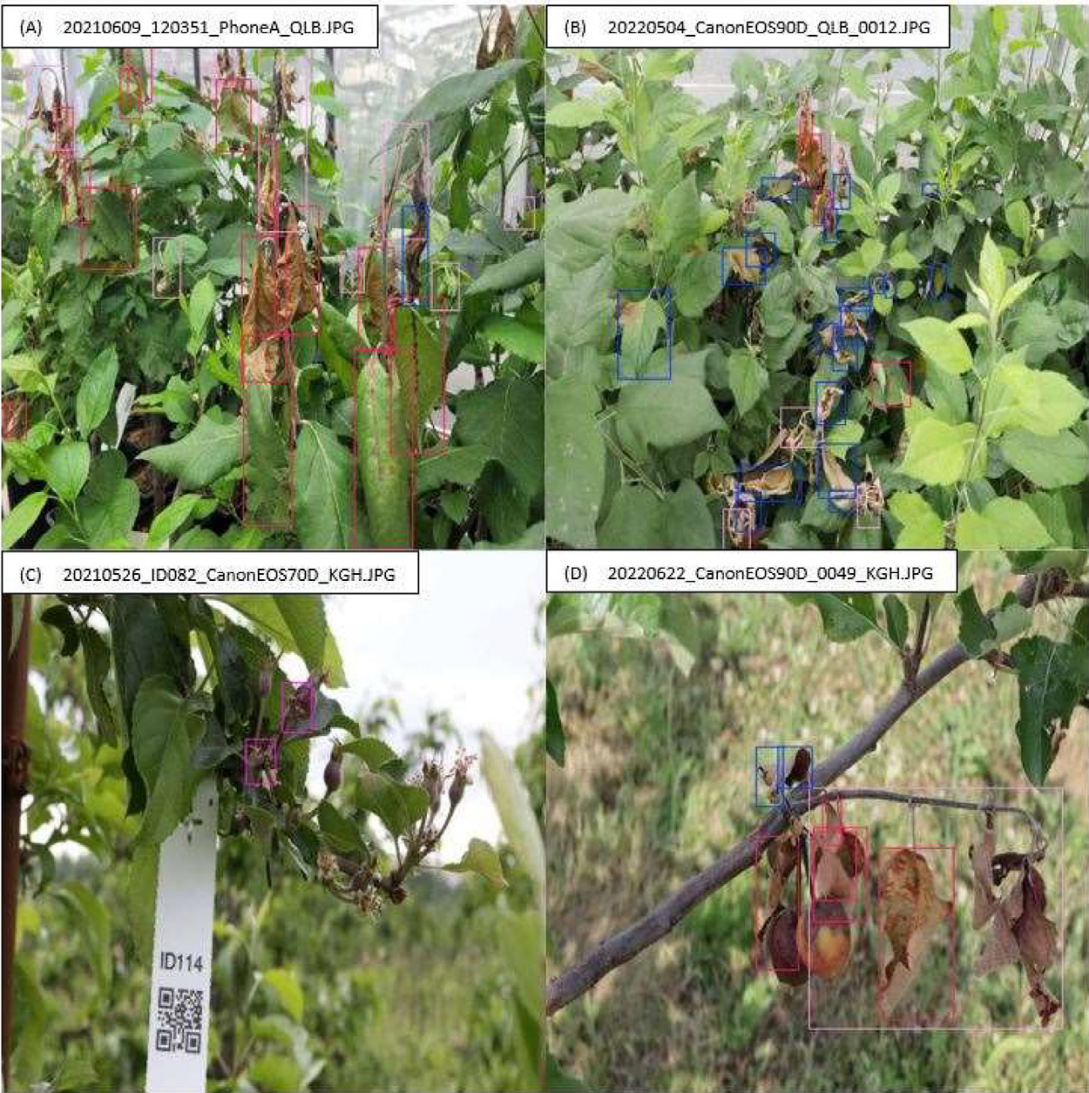
Examples of annotated images with fire blight infected leaves (red bounding boxes), flowers (purple bounding boxes) and shoots (rose bounding boxes), as well as the class ``MAYBE_'' (blue bounding boxes), used for the ERWIAM dataset. Images were recorded in the experimental greenhouse (Quedlinburg - QLB) in 2021 (A) and 2022 (B) and in the experimental orchard (Kirschgartshausen - KGH) in 2021 (C) and 2022 (D).

**Fig. 7:**
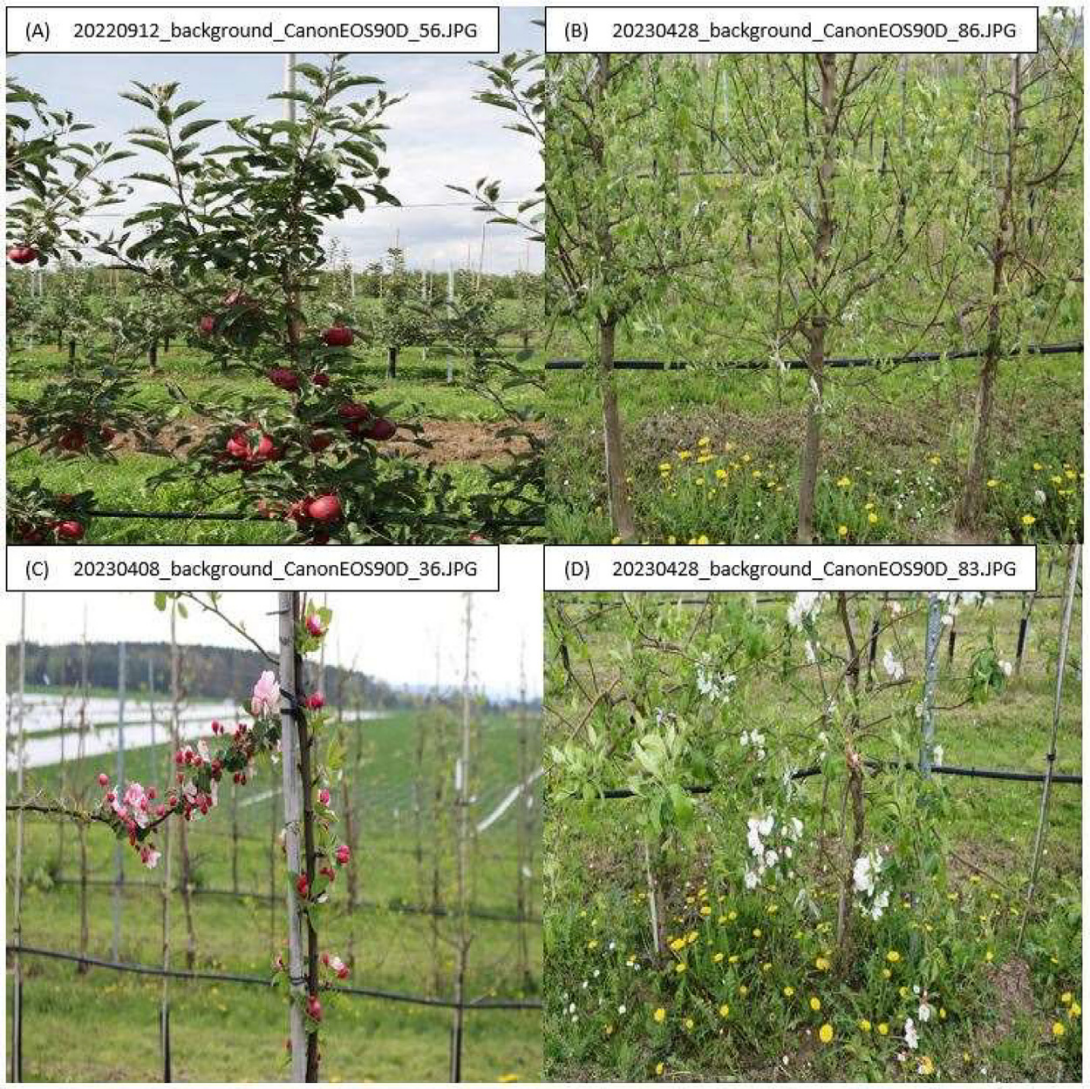
Examples of background images in 2022 (A) and 2023 (B–D) used in the ERWIAM dataset. Images were taken with the camera model Canon EOS 90D at the experimental orchard in Dresden–Pillnitz.

The one-stage detector model You Only Look Once (YOLO) was chosen because it recognises different objects very quickly and precisely, and requires less computing capacity than two-stage models due to its efficient training [17]. The images folder contains 1611 images with fire blight symptoms (Fig. 6) and 87 background images (Fig. 7).

## 4 Experimental Design, Materials and Methods

### 4.1 Data collection

The RGB raw data of fire blight symptoms were collected in 2021 and 2022 on different days in the experimental orchard and in the experimental greenhouse. The image data acquisition was carried out by employees of the Julius Kühn-Institute for breeding research on fruit crops, Leibniz Institute for Agricultural Engineering and Bioeconomy (ATB) and the company geo-konzept GmbH. Manual sorting was carried out with regard to image quality and the appearance of fire blight symptoms on flowers, leaves and shoots. For example, RGB images taken in the Kirschgartshausen and Quedlinburg trial sites that did not show any symptoms of disease were sorted out of the annotation dataset. In contrast, images taken in the Dresden-Pillnitz experimental orchard with only healthy leaves, flowers and shoots and no fire blight symptoms were sorted into the background images. To ensure that there were no fire blight symptoms in the background images, these were taken in Dresden-Pillnitz in 2022 and 2023 (Fig. 7).

A broad spectrum of symptoms of different genotypes was recorded and clear fire blight symptoms (classes ``FLOWER'', ``LEAF__'' and ``SHOOT_'') and possible fire blight symptoms (class ``MAYBE_'') were narrowed down. As a result, a large number of possible symptoms that could lead to false-positive results, but prevent potential fire symptoms from being overlooked, could be integrated into this dataset. Features in the image that are similar in colour and texture to fire blight symptoms were included. For example, the symptoms of branch breakage could lead to false-positive results and should be reduced by including the class ``MAYBE_'' and the background images.

After labelling, the size of the labelled images and the background images was reduced to 1280 × 1280 pixels with the help of the Pillow Python library using the Lanczos filter [15], as very different image sizes were available and the original images took up a lot of memory.

Different camera settings, varying distances to the objects or the multiple shooting angles are possibilities to create a diverse dataset. The light value of the different camera systems used to create the ERWIAM dataset ranged from +7.9 to +14.7 LV. The ISO varied from 25 to 6400, the shutter speed from 1/32 to 1/1203 and the f-number from 1.8 to 14.0 (Table 4).

Data acquisition under different weather and light conditions (Table 5) should also help to ensure robust recognition of the trained model. Cover levels of 7 to 8 octa were measured in the experimental orchard and a mean leaf wetness of 5 to 89 % on the recording days.

**Table 5:** Cloud cover [18,19] and the daily average of the leaf wetness [20] from 2021 up to and including 2023 in the experimental orchards in Kirschgartshausen (KGH) and Dresden-Pillnitz (DD).

Date	Location	Cloud cover c^,^^d^ [octa]	Average leaf wetness [%]
26/05/2021	KGH^a^	8	89
27/05/2021	KGH^a^	8	44
01/07/2021	KGH^a^	7–8	45
22/06/2022	KGH^a^	8	5
12/09/2022	DD^b^	7	–
08/04/2023	DD^b^	7–8	–
28/04/2023	DD^b^	8	–

a KGH: experimental orchard in Kirschgartshausen, JKI-Institute for Plant Protection in Fruit Crops and Viticulture in Dossenheim (Germany).

b DD: experimental orchard in Dresden-Pillnitz, JKI-Institute for Breeding Research on Fruit Crops in Dresden-Pillnitz (Germany).

c The cloud cover was recorded by the Mannheim weather station, which is located about 12 km southeast air distance of the experimental orchard in Kirschgartshausen.

d The cloud cover was recorded by the Dresden-Klotzsche weather station, which is located about 9 km north-west air distance of the experimental orchard in Dresden-Pillnitz.

### 4.2 Annotation work and data set

The CVAT labelling tool [8] and the 2-point bounding box labelling method were selected for the annotation. Rectangular bounding boxes were drawn around shoots, flowers and leaves. Prior to manual annotation, criteria for classifying the fire blight symptoms on the RGB images were compiled on the basis of the literature [[3], [4], [5]]:

Flower blight - class ``FLOWER'' (Fig. 1)

­ Discolouration of flower heads in brown-black with white-grey coating
­ Bacterial slime (milky-white to yellowish-orange) can emerge from the flower head and flower stalk

Symptomatic flower heads were annotated, without flower stalk. Green fruit mummies or green flowers were classified neither as a fire blight symptom, nor as ``MAYBE_''. Dark-coloured flower stalks could indicate an early fire blight infection, but were not annotated as a symptom of a flower infection.

Leaf blight - class ``LEAF__'' (Fig. 2)

­ Black or brown discolouration of the leaf veins: starting from the base of the blade or petiole and spreading over the midribs and lateral ribs
­ Bacterial slime (milky-white to yellowish-orange) may emerge from the petiole

Shoot blight - class ``SHOOT_'' (Fig. 3)

­ The Shepherd's Crook was annotated as the main characteristic
­ Shoot bends downwards in an arc shape
­ Initially still green to light green
­ Bacterial slime may appear
­ Shoots are later dry with a brown to black colouring

Sometimes a white/grey or light brown colouring of the shoot has been observed. However, a sole white/grey colouration or light brown colouration of a shoot was not classified as a clear sign of fire blight and was annotated as ``MAYBE_''. If the described leaf symptoms of fire blight also occurred, the infected shoot was annotated as ``SHOOT_'' together with the symptomatic leaves on the infected shoot. Within the ``SHOOT_'' bounding box, only leaves that exhibited the symptoms of the ``LEAF__'' class were annotated as ``LEAF__''. An attempt was made to record and annotate different perspectives of Shepherd's Crook.

Class ``MAYBE_'' (Fig. 4)

­ Leaves that showed no black or brown colouration from the base of the blade to the midrib and lateral rib
­ Flowers that showed a different colouring (e.g. light brown) from the symptoms described
­ Shoots that were clearly identified as branch breakage on the days of recording in the experimental orchard
­ Sole white/grey colouration or light brown colouration of a shoot

Overlapping and nested fire blight symptoms were also labelled (Fig. 6, sample A and D). Fire blight symptoms were also annotated if they showed light reflections or shading in addition to the infection (Fig. 1, Fig. 2, Fig. 3, 6, sample C and D) or were wet from rain.

It is also important to note that fire blight can spread quickly in orchards and cause devastating damage if it is not detected [[1], [2], [3], [4], [5], [6],^16^]. However, it is often not clearly recognizable on RGB images (as well as in nature), as the symptoms are similar to those of other pathogens and can still differ significantly depending on the genotype. Genetic analyses are required for a clear identification of *Erwinia amylovora* [[1], [2], [3],^6^]. To avoid overlooking unclear fire blight symptoms and thereby risking the spread of the disease in the orchard, the class "MAYBE_" was considered necessary in the ERWIAM dataset. This allows users to control the sensitivity to fire blight symptoms themselves when creating object detection models. Once the annotation work was completed, a labelling file (.txt) in YOLO 1.1 format was created for each image and used to train the YOLO model.

## Limitations

Not applicable.

## Ethics Statement

For this research and analysis, no human or animal subjects were used and no data from social media platforms was used. The authors confirm that the provided dataset and presented work strictly meet the ethics requirements for publication in Data in Brief as mentioned in https://www.elsevier.com/de-de/researcher/author/policies-and-guidelines.

## CRediT authorship contribution statement

**Virginia Maß:** Software, Methodology, Investigation, Validation, Data curation, Visualization, Writing – original draft. **Pendar Alirezazadeh:** Software, Methodology, Validation. **Johannes Seidl-Schulz:** Software, Validation, Methodology, Supervision, Visualization, Investigation. **Matthias Leipnitz:** Conceptualization, Methodology. **Eric Fritzsche:** Resources, Investigation. **Rasheed Ali Adam Ibraheem:** Software, Investigation. **Martin Geyer:** Supervision, Funding acquisition. **Michael Pflanz:** Conceptualization, Funding acquisition. **Stefanie Reim:** Project administration, Conceptualization, Funding acquisition, Supervision, Resources, Investigation, Validation, Visualization, Writing – review & editing.

## Data Availability

ERWIAM dataset (Original data) (Mendeley Data).

## References

[1] Bagheri N., Mohamadi-Monavar H., Azizi A., Ghasemi A. (2018). Detection of fire blight disease in pear trees by hyperspectral data. Eur. J. Remote Sens..

[2] Skoneczny H., Kubiak K., Spiralski M., Kotlarz J., Mikiciński A., Puławska J. (2020). Fire blight disease detection for apple trees: hyperspectral analysis of healthy, infected and dry leaves. Remote Sens..

[3] Peil A., Bus V., Geider K., Richter K., Flachowsky H., Hanke M.-V. (2009). Improvement of fire blight resistance in apple and pear. Int. J. Plant Breed., Global Sci. Books.

[4] van der Zwet T., Beer S.V., United States Agricultural Research Service, Cornell University (1999).

[5] Jarolmasjed S., Sankaran S., Marzougui A., Kostick S., Si Y., Quirós Vargas J.J., Evans K. (2019). High-throughput phenotyping of fire blight disease symptoms using sensing techniques in apple. Front. Plant Sci..

[6] Peil A., Emeriewen O.F., Khan A., Kostick S., Malnoy M. (2021). Status of fire blight resistance breeding in Malus. J. Plant Pathol..

[7] Mahlein A.-K. (2016). Plant disease detection by imaging sensors - parallels and specific demands for precision agriculture and plant phenotyping. Plant Dis.

[8] B. Sekachev, N. Manovich, M. Zhiltsov, A. Zhavoronkov, D. Kalinin, B. Hoff, T. Osmanov, D. Kruchinin, et al., opencv/cvat: v1.1.0, 2020, Zenodo, 10.5281/zenodo.4009388.

[9] P. Harvey, ExifTool, Kingston, Ontario, Canada, 2016, https://exiftool.org.

[10] Samsung Electronics Co., Ltd., Samsung Galaxy S10 (Version SM-G973F), 2019, https://imei.org/de/phone-model-lookup/samsung-galaxy-s10_7645.

[11] Samsung Electronics Co., Ltd., Samsung Galaxy S20 FE 5G (Version SM-G781B), 2020, https://imei.org/de/phone-model-lookup/samsung-galaxy-s20-fe-5g_7568.

[12] Samsung Electronics Co., Ltd., Galaxy Tab A 10.5 SM-T590. Sensor type: 3.6 mm × 2.8 mm CMOS, Effective pixels: approx. 8.0 megapixels, 2018, https://www.samsung.com/de/business/tablets/galaxy-tab-a/galaxy-tab-a-t590-sm-t590nzkadbt.

[13] Canon, Canon Inc (2013). https://www.canon-europe.com/for_home/product_finder/cameras/digital_slr/eos_70d/specifications/.

[14] Canon Inc., EOS 90D. Sensor type: 22.3 mm × 14.8 mm CMOS, Effective pixels: approx. 32.50 megapixels, Canon, Tokyo, Japan, 2019, https://www.canon-europe.com/cameras/eos-90d/specifications.

[15] J.A. Clark, Pillow (PIL Fork) Documentation (Version 9.5.0), readthedocs, 2023, https://pillow.readthedocs.io/en/stable/releasenotes/9.5.0.html.

[16] Billing E., Berrie A.M. (2002). A Re-examination of fire blight epidemiology in England. Acta Hortic..

[17] Redmon J., Divvala S., Girshick R., Farhadi A. (2016). 2016 IEEE Conference on Computer.

[18] Deutscher Wetterdienst (DWD), Hourly station observations of cloudiness for Germany, recent, Version v23.3, Dresden-Klotzsche (1048), 2023, https://opendata.dwd.de/climate_environment/CDC/observations_germany/climate/hourly/cloudiness/recent/stundenwerte_N_01048_akt.zip.

[19] DWD Climate Data Center (CDC), Stündliche Stationsmessungen des Bedeckungsgrades in Achtel für. Version v21.3, https://cdc.dwd.de/portal/202209231028/mapview.

[20] Agrarmeteorologie Baden-Württemberg, Wetterstation Kirschgartshausen: tagesmittelwerte des Monats, abgerufen am 2024, https://www.wetter-bw.de/Internet/AM/NotesBwAM.nsf/(XP_StationABC_All)/8457ec17ea683253c12581f4002e864b?OpenDocument&TableRow=3.1.1,3.4#3.1.

